# A Study to See the Effect of Social Media Usage Among Healthcare Providers

**DOI:** 10.7759/cureus.16350

**Published:** 2021-07-13

**Authors:** Mohammad Noah Khan, Ahmad Faraz, Abdul Basit Jamal, Sarah Craig, Waqas Ilyas, Fatima Ahmad, Muhammad Jamshed, Waleed Riaz

**Affiliations:** 1 Trauma and Orthopaedics, Royal Victoria Hospital, Belfast, GBR; 2 Trauma and Orthopaedics, Leeds Teaching Hospitals NHS Trust, Leeds, GBR; 3 Trauma and Orthopaedics, Ittefaq Hospital (Trust), Lahore, PAK; 4 Cardiology, Royal Victoria Hospital, Belfast, GBR; 5 Orthopaedics, Southampton General Hospital NHS Foundation Trust, Southampton, GBR; 6 Anesthesia, Punjab Institute of Cardiology, Lahore, PAK; 7 Internal Medicine, Shrewsbury & Telford Hospital NHS Trust, Shrewsbury, GBR; 8 Surgery, Royal Victoria Hospital, Belfast, GBR

**Keywords:** social media, healthcare workers, nurses, policy, patient centered care

## Abstract

Purpose

This study aimed to assess how healthcare professionals (HCPs) use social media to determine how it influences the quality of patient care.

Materials and methods

This is a cross-sectional study conducted over eight months, between August 2020 and March 2021 using a questionnaire and checked amongst investigators.

Results

One hundred fifty-eight participants had electronic devices and 145 (91.9%) used social media at work. 26.6% of these HCPs said they spent less than an hour on social media forums, 31% said they spent one to two hours, 28.5% said two to three hours, and 13.9% said they spent more than four hours. As compared to nurses (46%), consultants and pharmacists use social media at a much lower rate (1% for each group). Compared to junior doctors, a higher percentage of nurses (40%) said they were aware of a social media policy at their hospital (8%). A quarter of healthcare employees (20%) were unaware of their workplace policy, potentially exposing sensitive medical details to the public. More research is needed to assess the particular effects of these results on patient care quality and can help in providing literature informing applications encrypted and secure patient data.

Conclusion

According to our results, a large percentage of healthcare quality professionals used social media networks. A significant proportion of doctors and nurses use it to visit online medical forums for improving education. A large portion of surveyed sample was unaware of hospital policy on social media usage. Further education is required to improve the right use of social media in the hospital setting.

## Introduction

Previous studies on social media utilisation in healthcare recognise impacts of social media utilize by patients for wellbeing-related reasons inside the healthcare framework. Social media can serve as a help to patients [1]. Similarly, the advance of online technologies will also support practitioners in collecting, managing, and interacting for higher quality service. However, poor quality of information, patient confidentiality, and legal issues are some risks and challenges that could impact the effective and beneficial integration of online platforms [1].

Studies have analysed the rate of social media adoption and usability from the point of view of different stakeholders. In medical education, a study found that the degree of popularity and awareness is higher in undergraduate than in postgraduate circles. However, the study revealed that both groups had an interest in the use of new technologies [2].

Adopting online platforms by doctors is increasing; however, guidelines and policies may act as barriers and reduce the quantity of information posted or shared to protect patient confidentiality [3]. 

The new online media are changing the communication behaviours of various stakeholders and increasing the exchange of information among healthcare professionals (HCPs) for an optimal decision process. However, the value of patient privacy should be respected on highly interactive and open platforms [4].

The use of social media within a healthcare context is affected by ethical dilemmas and privacy concerns that could prevent users from benefiting from this highly interactive means of communication. Research and trials are trying to explain the mechanism of information transfer and interaction by different users [5].

Practitioners in different healthcare disciplines can benefit from the significant data generated from the patients’ interaction within the social media groups. Moreover, interpersonal communication can be improved, and evidence-based knowledge can be diffused faster than traditional channels. Research might explore the safe and cost-effective use of social media to complement the evidence-based practice; moreover, the policies and guidelines can support the efficient adoption of personal development and knowledge updates [6].

The identification of the factors that promote practical use along with the effective type of platforms represents a knowledge gap and requires further research [7].

The preliminary evidence of adopting social media in different healthcare practices reveals divergent opinions on the benefits, challenges, level of information exchange, communication, and productivity achieved. The study of users’ characteristics, behaviours, and external factors that relate to usage intention and frequency of use represent the potential to advance knowledge on the optimal integration of social media in healthcare practice. 

The primary research we have collected data is to understand the relationships between the influential factors and the adoption and usage of social media by HCPs. The research also aims to understand the strength of relationships between the underlying factors and the adoption of social media by HCPs.

## Materials and methods

This is a cross-sectional study conducted in a major trauma centre in the Department of Trauma & Orthopedics, where health professionals of all categories such as band 6-8 nurses, junior doctors, consultants and junior staff were included. This study was conducted over a time span of 8 months, where data was collected between August 2020 to March 2021 using a questionnaire, which was reviewed amongst investigators using the previously designed study [8]. After getting this study approved as a quality improvement project with clinical governance, the individuals were given a questionnaire and were asked to fill and return it the next day. The health care professionals who did not fill the questionnaire properly or refused to fill were excluded from the study. Data were manually entered into SPSS after collection from the participants and analysed electronically. Data analysis was done using the Statistical Package for the Social Sciences (SPSS), version 23 (IBM Inc., Armonk, NY)

## Results

A total of 158 questionnaires were filled by the candidates. There were 41 male participants (25.9%), 116 female participants (73.4%), and one participant who did not want to react (0.6%). Every one of the 158 participants had an electronic device, and 144 (91.1%) of them used social media forums (Table 1).

**Table 1:** Baseline characteristics of participants in the study including age, gender, job.

Indicator	Category	Frequency (N)	Percentage (%)
Gender	Female	116	73.4
	Male	41	25.9
	Other	1	0.6
Age group	21-30 Y	79	50.0
	31-40 Y	37	23.4
	41-50 Y	18	11.4
	51-60 Y	23	14.6
	61-70 Y	1	0.6
Job category	Consultant	3	1.9
	Junior Doctor	37	23.4
	Nurse	73	46.2
	Healthcare Assistant	22	13.9
	Pharmacist	2	1.3
	Other	21	13.3

Out of 158 participants, 79 (50%) were between the ages of 21 and 30, and 37 (23%) were between the ages of 31 and 40. 26.6 percent of healthcare providers said they spent less than an hour on social media forums, 31% said they spent one to two hours, 28.5% said two to three hours, and 13.9% said they spent more than four hours during the course of a day (see Table 2).

**Table 2:** Association between use of social media forums and different age groups of healthcare workers.

	21-30 Y	31-40 Y	41-50 Y	51-60 Y	61-70 Y
Do you use social media?	78	34	16	15	1
Do you think you waste your time on social media?	49	24	12	9	0
Do you use social media before going to bed?	76	28	11	10	0
Do you think social media takes away from your family time?	35	15	9	8	1
Do you get less sleep because of using social media?	32	4	2	3	0
Do you think social media forums have correct Info?	4	1	1	0	0

Pearson chi-square test was used to check the association between the use of social media forums and different age groups of healthcare workers.45.5% of regular social media users say they use it for work-related tasks like medical reading, while 11% say they link to online medical forums (Table 3). The use of social media is considered a waste of time by 59% of the participants (Table 2)

**Table 3:** Association between use of social media forums and different job categories of healthcare workers with P-value calculated by Pearson chi squared test show a relationship between categorical variables.

	Consultant	Junior Doctor	Nurse	Healthcare Assistant	Pharmacist	Other	P-value
Do you use social media for Medical Reading?	1	21	32	9	0	9	0.54
Do you contribute to medical forums online?	0	3	9	2	0	3	0.92
Do you think information on social media is correct?	0	2	2	1	0	1	0.52
Do you recommend your patients to search for illnesses online?	1	8	7	2	0	2	0.39
Are you aware of workplace social media policy?	2	25	63	17	2	17	0.60

Regarding the hospital's institutional social media policy, 79.7% of subjects were aware of its presence. Just 3.7% of participants thought the information they received on social media was reliable, and only 12.6% would allow patients to use social media for medical purposes.

In the sample population, there were differences in the usage of social media between physicians (n = 3) and nurses (n = 73). (Table 1). Physicians consisting of consultants and junior doctors spent far less time on social media than nurses (Table 2). Our study found that 8.1% of junior doctors and 12.3% of nurses contributed to online medical forums. Just 9.5% of nurses advised their patients to check online social media and medical forums to check for information about their illnesses and disease processes, while 21.6% of junior doctors did so. 67.5% of junior doctors were aware of the hospital's social media practices of the hospital, compared to 86.3% of nurses.

## Discussion

Social media is one of the most disruptive business forces to emerge in recent years [8]. However, social media may have negative consequences that should be addressed, especially when it comes to patient confidentiality. In terms of community engagement, health promotion, patient education, outreach, and other factors, the use of social media in healthcare settings is growing on a daily basis [9]. With all of the advantages of being able to access medical records via various websites or applications, social media can be the source of distraction.

Moqbel et al. [10] found that every day for 15 minutes on Facebook, job performance was reduced by 1.5% in a healthcare setup. The current study suggests that nearly 55% of health care providers use social media for one to four hours every day while being at work.

James Brown et al. [11] found that 75% of healthcare workers used social media while being at work, the present study recorded that 91% of medics used social media during their work, whereas another study found that 87% of health professionals used social media [7]. Online media can likewise be a huge interruption in the working environment. With every one of the advantages of getting to clinical records effectively through various sites or applications [12].

Junior doctors and nurses were noted to be maximally using social media on a day-to-day basis. A study published in 2014 showed that young physicians have grown up with online correspondence, therefore the daily usage of social media is with higher frequency. In contrast, senior specialists have not been as engaged with online media into day-by-day life, nor the expanding volume of its utilization, validating our findings where the P-value was found to be <0.001 [11]. Social media is all around; its utilization has developed dramatically over the late years. The predominance of these sources for correspondence raises some fascinating and possibly dangerous issues for doctors. De Camp et al. .published a study in 2013 which proposed that in a time-pressured day, healthcare workers should not spend time on tweets or social media posting, the present study doesn’t agree with the above-mentioned recommendations where the P-value is found to be 0.09 [13], this could be off the reason that they use the internet to read medical-related stuff.

This survey shows that health care professionals quite often use social media before going to bed (P-value: 0.00). Sleep disturbances and deficient rest span are related to daytime sleepiness and a scope of chronic weakness results [14]. Jessica et al. [15] found a strong association between lack of sleepiness and use of social media in a subject size of 1788, we recorded similar results where our health care professionals reported sleep-related problems (P-value: 0.00). Turki Alanzi et al. [16] found that 30.6% of the medical professionals think that it isn't advisable to look through online data about patients, and 44.3% of them thought that patients would not believe the clinical guidance if a doctor got the data from a site. The current study found that fewer number of hospital staff would use social media to educate themselves, where P-value was found to be 0.54. Furthermore, they would not recommend medical information to be believed by their patient, which has been discussed on various social forums.

Surani et al. [7] found that 40% of healthcare professionals are not aware of the institutional policy regarding the use of online media, which could cause a security penetrate of classified clinical data. We found that our large proportion of workers were not aware of hospital policy regarding the use of social media which was recorded as 80% (P-value: 0.60). This is troubling because understanding institutional policies with regards to online media is essential in ensuring private clinical data.

Limitations

The small sample size of a single hospital limits the applicability of the findings to a wider population. Variables such as marital status, family life and affect of children can also be explored. Furthermore, cultural and geographic differences among the community's population may restrict the generalizability of this article, necessitating similar studies in multiple hospitals. Furthermore, using a standardized questionnaire in a multicenter study will aid in the collection of data from a larger sample size. 

## Conclusions

According to our findings, doctors contribute less to online medical forums than nurses. Since they do not believe the information on social media (Facebook, Twitter ) is accurate, a limited number of physicians and nurses advised their patients to learn about their disease processes online. Our study shows that social media was used by a significant percentage of healthcare quality professionals in the United Kingdom. It is used by a large number of doctors and nurses to access online medical forums in order to further their education. According to the results, social networking may be useful in deciding healthcare efficiency in the United Kingdom, and literature may help in the creation of encrypted applications for sharing patient-sensitive data.
